# ^18^ F-FDG PET/CT in Cardiac Sarcoidosis: Pioneering Evidence from the Arab Middle East

**DOI:** 10.1055/s-0046-1824539

**Published:** 2026-06-17

**Authors:** Joe Rizkallah, Omar El Sardouk, Safaa Al Zakleet, Nina Saliba, Marwan Refaat, Habib Dakik, Diana Paez, Thomas N. B. Pascual, Enrique Estrada-Lobato, Francesco Giammarile, Alain S. Abi-Ghanem, Mohamad Haidar

**Affiliations:** 1 Department of Diagnostic Radiology, American University of Beirut, Beirut, Lebanon; 2 Division of Cardiology, Department of Internal Medicine, American University of Beirut, Beirut, Lebanon; 3 Nuclear Medicine and Diagnostic Imaging Section, Division of Human Health, Department of Nuclear Sciences and Applications, International Atomic Energy Agency, Vienna, Austria; 4 Philippine Nuclear Research Institute, Quezon City, Philippines

**Keywords:** cardiac sarcoidosis, positron emission tomography, multimodal imaging, nuclear medicine, Middle East

## Abstract

**Objectives:**

Cardiac sarcoidosis can lead to arrhythmias, heart failure, and sudden cardiac death. Positron emission tomography/computed tomography (PET/CT) is crucial for diagnosis and monitoring, yet data from the Middle East and North Africa (MENA) region remain scarce. This study evaluated the role of PET/CT in diagnosing and managing cardiac sarcoidosis at a tertiary referral center in the Middle East.

**Materials and Methods:**

This retrospective study included 19 patients with biopsy-proven sarcoidosis who underwent fluorine-18 fluorodeoxyglucose (FDG) PET/CT for suspected cardiac involvement at a tertiary referral medical center in Lebanon between 2014 and 2024. Complementary imaging with echocardiography, cardiac magnetic resonance imaging, and electrocardiography was also analyzed alongside treatment approaches and follow-up outcomes.

**Results:**

FDG PET/CT identified cardiac involvement in 12 patients, with diffuse or focal FDG uptake patterns correlating with symptom severity and electrocardiographic abnormalities. These patients exhibited higher rates of arrhythmias, conduction abnormalities, and reduced global longitudinal strain and E/A ratio on echocardiography. Cardiac magnetic resonance imaging demonstrated late gadolinium enhancement in most cases with FDG PET/CT-confirmed cardiac sarcoidosis, supporting the presence of myocardial inflammation. Treatment included corticosteroids, immunosuppressive agents, and device implantation in selected cases. Follow-up FDG PET/CT showed significant reductions in FDG uptake, indicating therapeutic response.

**Conclusion:**

This study underscores the clinical utility of FDG PET/CT in diagnosing and managing cardiac sarcoidosis, particularly in resource-limited settings like the MENA region. FDG PET/CT enabled early detection, guided treatment decisions, and facilitated monitoring of therapeutic response. These findings highlight the need for broader access to FDG PET/CT imaging in the region to optimize patient outcomes.

## Introduction

Sarcoidosis is a systemic inflammatory disease characterized by granuloma formation in multiple organs, most commonly the lungs. 1 It typically develops before the age of 50 years and is more prevalent among women, African American populations, and in regions such as Scandinavia and North America. 1 2 Clinical presentation and severity vary depending on the involved sites, complicating diagnosis and management. In particular, cardiac involvement is often missed, leading to increased morbidity and mortality if untreated. 3

Cardiac sarcoidosis involves granulomatous inflammation affecting the heart, which may result in arrhythmias, heart failure, and sudden cardiac death. 3 The clinical diagnosis of cardiac sarcoidosis remains challenging in light of its variable and nonspecific symptoms. 4 Imaging modalities such as magnetic resonance imaging (MRI), echocardiography, and positron emission tomography/computed tomography (PET/CT) are essential for diagnosis and monitoring. Among these, fluorine-18 fluorodeoxyglucose (FDG) PET/CT is particularly valuable for detecting active inflammation and assessing treatment response. 4

This study evaluated the role of FDG PET/CT in diagnosing and managing cardiac sarcoidosis at our institution, a tertiary referral center in the Middle East. It examined the characteristics of patients diagnosed and followed up with FDG PET/CT, aiming to clarify how FDG PET/CT aided early detection, accurate diagnosis, and optimal management of cardiac sarcoidosis, ultimately improving patient outcomes in this resource-limited region.

## Materials and Methods

### Study Design and Patient Population

This retrospective descriptive study was conducted at our institution, focusing on patients undergoing FDG PET/CT for suspected cardiac sarcoidosis. Eligibility criteria included a suspected diagnosis of cardiac sarcoidosis based on medical history, clinical presentation, and/or imaging findings. Patients were included if they underwent cardiac FDG PET/CT. Extracardiac FDG PET/CT, myocardial perfusion imaging, electrocardiography (EKG), echocardiography, and cardiac MRI results were also examined when available. Patients with inadequate clinical documentation were excluded.

### Data Collection

The study was approved by the institutional review board, and informed consent was obtained from all patients for the use of clinical and imaging data. The study adhered to the principles of the Declaration of Helsinki. Patient records from 2014 to 2024 were reviewed to collect demographics (age, sex), clinical presentation (symptoms, prior history of sarcoidosis), EKG results, and imaging findings from FDG PET/CT, echocardiography, and cardiac MRI. FDG PET/CT findings were categorized as FDG uptake patterns (diffuse, focal, or absent), with documentation of specific involvement sites. Additional data included therapeutic regimens, imaging changes on follow-up, and treatment response.

### Statistical Analysis

Continuous variables were expressed as mean ± standard deviation, whereas categorical variables were summarized as frequencies and percentages. Given the relatively small sample size and nonnormal distribution of several imaging parameters, nonparametric statistical tests were used for exploratory analyses. Comparisons between groups were performed using the Mann–Whitney U test or Kruskal–Wallis test for continuous variables and Fisher's exact test for categorical variables, as appropriate. Paired comparisons of cardiac SUVmax before and after treatment were performed using the Wilcoxon signed-rank test. Exploratory correlations between imaging and echocardiographic parameters were assessed using Spearman correlation coefficients. Statistical significance was defined as *p* <0.05. Statistical analyses were performed using SPSS version 25 (IBM Corp., Armonk, New York, United States).

## Results

Table 1 summarizes patient characteristics, imaging findings, and treatment outcomes, highlighting key demographic and clinical insights. Table 2 provides an overview of relevant findings, emphasizing the diagnostic and therapeutic impact of advanced imaging in cardiac sarcoidosis. Table 3 presents exploratory statistical comparisons between patients with negative cardiac FDG PET/CT findings, focal uptake, and diffuse uptake.

**Table 1:** Clinical, imaging, and treatment characteristics of patients with suspected cardiac sarcoidosis

Patient	Age	Sex	EKG findings	Echocardiographic findings	Cardiac PET/CT findings	Cardiac SUVmax	Cardiac MRI findings	Treatment regimen	Clinical follow-up outcome	Cardiac PET/CT follow-up findings	Cardiac SUVmax follow-up
1	70	Male	PVCs	LVEF: 35–39%GLS: −14.3%E/A ratio: 0.67Moderate global hypokinesia of the LV.	Diffuse increased FDG uptake throughout the left ventricular myocardium, predominant in the anterior and lateral walls.	8.6	LVEF: 30%RVEF: 41% LGE: present	Prednisone and methotrexate + treatment for HFrEF	Asymptomatic	Resolution of radiotracer uptake, in keeping with good response to treatment.	3.0
2	37	Male	PACs	LVEF: 45–49%GLS: −15.8%E/A ratio: 1.11	Diffuse increased FDG uptake throughout the left ventricular myocardium, with mild decreased uptake at the apex.	6.2	LVEF: 43%RVEF: 40% LGE: present	Prednisone, methotrexate, and infliximab	Asymptomatic	Resolution of radiotracer uptake, in keeping with good response to treatment.	4.1
3	41	Male	PACs and PVCs	LVEF: 45–49%GLS: −14.7%E/A ratio: 0.76	Diffuse increased FDG uptake throughout the left ventricular myocardium, most marked in the lateral wall at the base.	12.2	LVEF: 42% RVEF: 47% LGE: present	Prednisone and infliximab	Asymptomatic	Resolution of the radiotracer uptake, in keeping with good response to treatment.	3.5
4	71	Male	Left anterior fascicular block, right bundle branch block, PVCs, PACs	LVEF: 45–49%GLS: −13.8%E/A ratio: 1.15 Akinesia of the inferobasal segments, grade II diastolic dysfunction, and moderate pulmonary hypertension.	Diffuse increased FDG uptake throughout the left ventricular myocardium, mainly at the level of the anterior and inferior walls.	7.5	N/A	Deflazacort and methotrexate + ICD placement	Worsening fatigue, dyspnea, and chest discomfort + drop in LVEF to 35-39%	Persistent diffuse increased FDG uptake throughout the left ventricle.	8.0
								Prednisone and azathioprine + treatment for HFrEF	Asymptomatic	Resolution of the radiotracer uptake, in keeping with good response to treatment.	4.2
5	36	Male	Sinus bradycardia	LVEF: 60–64%GLS: −14.9%E/A ratio: 1.08	Diffuse increased FDG uptake in the left ventricular wall, most marked in the lateral wall.	6.4	N/A	Prednisone and hydroxychloroquine	Worsening fatigue, dyspnea, and chest discomfort + stable echocardiographic findings	Diffuse increased FDG uptake in the left ventricular wall, now involving all the walls.	7.8
								Infliximab	Asymptomatic	Resolution of the radiotracer uptake, in keeping with good response to treatment.	3.7
6	54	Male	Normal	LVEF: 60–64%GLS: −15.2%E/A ratio: 1.02	Diffuse increased FDG uptake throughout the left ventricular myocardium.	5.6	LVEF: 50%RVEF: 38% LGE: absent	Prednisone and methotrexate	Asymptomatic	Resolution of radiotracer uptake, in keeping with good response to treatment.	4.0
7	41	Female	PVCs	LVEF: 60–64%GLS: −19.5%E/A ratio: 1.13	Focal area of increased FDG uptake in the anterior wall of the left ventricular myocardium, at the level of the base.	6.1	LVEF: 52%RVEF: 41% LGE: present	Prednisone	Asymptomatic	Resolution of radiotracer uptake, in keeping with good response to treatment.	3.8
8	48	Female	Normal	LVEF: 55–59%GLS: −19.3%E/A ratio: 0.68	Focal areas of increased FDG uptake involving the anteroseptal and inferolateral cardiac walls.	16.4	N/A	Prednisone and azathioprine	Asymptomatic	Resolution of radiotracer uptake, in keeping with good response to treatment.	4.3
9	49	Male	Normal	LVEF: 55 − 59%GLS: −20.9%E/A ratio: 0.83	Focal areas of increased FDG uptake in the lateral and lower septal cardiac walls.	6.3	N/A	Prednisone	Asymptomatic	Resolution of radiotracer uptake, in keeping with good response to treatment.	3.6
10	55	Male	Bradycardia with complete atrioventricular block	LVEF: 55 − 59%GLS: −18.2%E/A ratio: 0.77	Focal area of increased FDG uptake in the interventricular septum extending to the free wall of the right ventricle.	17.7	N/A	Prednisone + permanent pacemaker placement	Asymptomatic	Resolution of radiotracer uptake, in keeping with good response to treatment.	3.9
11	33	Male	Ventricular tachycardia	LVEF: 60 − 64%GLS: −18.3%E/A ratio: 1.18	Focal areas of increased FDG uptake involving the interventricular septum, anteroseptal and septal walls extending from base to mid, left ventricular apex, and right lateral ventricular wall.	11.4	LVEF: 54%RVEF: 38% LGE: present	Prednisone and adalimumab + ICD placement	Asymptomatic	Resolution of radiotracer uptake, in keeping with good response to treatment.	3.2
12	43	Male	Normal	N/A	Focal areas of increased FDG uptake involving the mid and basal septum, lateral wall, and apex.	8.2	N/A	Prednisone	Asymptomatic	Resolution of radiotracer uptake, in keeping with good response to treatment.	4.1
13	57	Male	Normal	LVEF: 55–59%GLS: −21.3%E/A ratio: 1.14	No radiotracer-avid uptake along the myocardial walls to suggest cardiac sarcoidosis.	2.1	LVEF: 50%RVEF: 49% Increased signal with delayed enhancement in the epicardial layer of the mid and apical inferior and lateral walls, suggestive of old myocarditis.	Prednisone and infliximab	Asymptomatic	N/A	N/A
14	48	Female	Normal	N/A	No radiotracer-avid uptake along the myocardial walls to suggest cardiac sarcoidosis.	2.5	N/A	Prednisone	Asymptomatic	N/A	N/A
15	57	Female	Normal	LVEF: 60–64%GLS: −18.9%E/A ratio: 1.03	No radiotracer-avid uptake along the myocardial walls to suggest cardiac sarcoidosis.	3.2	N/A	Prednisone	Asymptomatic	N/A	N/A
16	34	Female	Normal	LVEF: 60–64%GLS: −20.5%E/A ratio: 1.16	No radiotracer-avid uptake along the myocardial walls to suggest cardiac sarcoidosis.	2.9	LVEF: 59% RVEF: 49% No foci of delayed enhancement.	Prednisone and methotrexate	Asymptomatic	N/A	N/A
17	62	Female	Sinus bradycardia	LVEF: 60–64%GLS: −20.9%E/A ratio: 1.00	No radiotracer-avid uptake along the myocardial walls to suggest cardiac sarcoidosis. Significantly decreased FDG uptake in the mid anterior left ventricular wall extending to the septum of the ventricular wall, in keeping with an old infarct.	2.7	LVEF: 58%RVEF: 46%No foci of delayed enhancement.	Prednisone and methotrexate + treatment for coronary microvascular spasm	Asymptomatic	N/A	N/A
18	32	Male	Normal	N/A	No radiotracer-avid uptake along the myocardial walls to suggest cardiac sarcoidosis.	3.0	N/A	Prednisone	Asymptomatic	N/A	N/A
19	33	Male	Normal	N/A	No radiotracer-avid uptake along the myocardial walls to suggest cardiac sarcoidosis.	2.6	N/A	Prednisone	Asymptomatic	N/A	N/A

Abbreviations: EKG, electrocardiography; FDG, fluorodeoxyglucose; GLS, global longitudinal strain; HFrEF, heart failure with reduced ejection fraction; ICD, implantable cardioverter-defibrillator; LGE, late gadolinium enhancement; LVEF, left ventricular ejection fraction; MRI, magnetic resonance imaging; PAC, premature atrial contraction; PET/CT, positron emission tomography/computed tomography; PVC, premature ventricular contraction; RVEF, right ventricular ejection fraction.

**Table 2:** Key diagnostic findings and treatment outcomes in cardiac sarcoidosis

Category	Key findings
Cardiac PET/CT	PET/CT revealed cardiac involvement in 63% of patients with suspected cardiac sarcoidosis, split evenly between diffuse and focal FDG uptake patterns.
Myocardial perfusion imaging	Diffuse FDG uptake often corresponded to normal perfusion, while focal uptake was associated with hypoperfusion.
EKG	Arrhythmias were common, correlating with PET/CT findings of active inflammation.
Echocardiography	Patients with cardiac sarcoidosis showed reduced GLS and E/A ratios, indicating myocardial dysfunction.
Cardiac MRI	LGE was prominent in cardiac sarcoidosis cases, reflecting active inflammation.
Treatment outcomes	Steroid-based therapy combined with immunosuppressants was effective in all patients, with marked improvement in SUVmax.
Follow-up insights	PET/CT demonstrated its utility in monitoring treatment response, with posttreatment SUVmax reduction.
Regional implications	The study highlighted the importance of PET/CT in resource-limited settings like the MENA region for improved outcomes.

Abbreviations: EKG, electrocardiography; FDG, fluorodeoxyglucose; GLS, global longitudinal strain; LGE, late gadolinium enhancement; MENA, Middle East and North Africa; MRI, magnetic resonance imaging; PET/CT, positron emission tomography/computed tomography.

**Table 3:** Exploratory statistical comparison of imaging, echocardiographic, and rhythm findings according to cardiac PET/CT involvement pattern

Parameter	Negative PET/CT	Focal uptake	Diffuse uptake	*p* -Value
GLS (%)	−20.4 ± 1.1	−19.2 ± 1.1	−14.8 ± 0.7	0.004
E/A ratio	1.1 ± 0.1	0.9 ± 0.2	0.9 ± 0.2	0.577
Mean cardiac SUVmax	2.7 ± 0.4	11.0 ± 4.8	7.8 ± 2.4	0.002
Reduced LVEF (<50%)	0/4	0/5	4/6	0.017
Arrhythmias/conduction abnormalities	1/7	3/6	5/6	0.045
Correlation between SUVmax and GLS	Spearman rho = 0.56	–	–	0.029

Abbreviations: GLS, global longitudinal strain; LVEF, left ventricular ejection fraction; PET/CT, positron emission tomography/computed tomography; SUVmax, maximum standardized uptake value.

Note: Exploratory analyses were performed using Kruskal–Wallis and Fisher's exact tests owing to the small subgroup sizes. Correlation analysis was performed using Spearman rank correlation.

### Patient Characteristics

Nineteen patients were included, with a mean age of 47 ± 12 years (range: 32–71 years). Thirteen were male, and six were female. All patients had biopsy-proven sarcoidosis with extracardiac involvement in the lungs and thoracic lymph nodes. In addition, three had abdominal involvement, two had bone involvement, one had ocular involvement, and one had skin involvement. All patients were symptomatic to varying extents at presentation, most commonly reporting fatigue, dyspnea, chest discomfort, palpitations, and lightheadedness.

### FDG PET/CT Imaging Findings

Seven patients showed no myocardial radiotracer uptake suggestive of cardiac sarcoidosis, whereas 12 had findings consistent with cardiac involvement. Notably, patients with negative cardiac FDG PET/CT results had a minimal burden of symptoms compared to those with findings of cardiac sarcoidosis, suggesting lower disease activity. For instance, patients with negative cardiac imaging tended to have occasional mild fatigue or dyspnea only upon severe exertion, compared to significant and persistent fatigue or dyspnea at rest in patients with cardiac sarcoidosis.

Among the seven patients with negative cardiac imaging, two also had no significant extracardiac findings, suggesting successful treatment and remission. The remaining five had FDG-avid extracardiac lesions, primarily in mediastinal and hilar lymph nodes, indicating localized disease.

Among the 12 patients with cardiac involvement, six exhibited diffuse FDG uptake throughout the left ventricular myocardium, whereas the other six had focal uptake, predominantly involving the interventricular septum and focal areas of the left ventricular wall. The mean SUVmax of cardiac lesions was 9.4 ± 4.2. No significant differences in extracardiac findings were noted between diffuse and focal uptake groups; in each group, two patients had no extracardiac disease, while four had FDG-avid extracardiac uptake, mostly in mediastinal and hilar lymph nodes, with some showing abdominal lymph node and bone involvement.

Patients with diffuse cardiac involvement had normal perfusion imaging without significant perfusion defects, whereas those with focal FDG uptake showed corresponding hypoperfusion in the affected areas. For example, one patient with diffuse uptake had normal perfusion, whereas another patient with focal FDG uptake in the mid and basal septum, lateral wall, and apex exhibited hypoperfusion in those areas.

### EKG Findings

All patients underwent resting EKG and 24-hour Holter monitoring. Among the seven patients with negative cardiac FDG PET/CT findings, six had normal EKGs, whereas one had sinus bradycardia related to coronary microvascular spasm rather than cardiac sarcoidosis. Among patients with focal cardiac FDG uptake, three had normal EKGs, whereas the remaining patients demonstrated significant conduction or rhythm abnormalities, including complete atrioventricular block, ventricular tachycardia, and frequent premature ventricular contractions (PVCs) exceeding 1,000 per day. Patients with diffuse cardiac FDG uptake showed the greatest arrhythmic burden; five of six had EKG abnormalities, including sinus tachycardia with frequent PVCs/premature atrial contractions (PACs), bundle branch block, fascicular block, or sinus bradycardia. Overall, arrhythmias and conduction abnormalities were more frequent in patients with positive cardiac FDG PET/CT findings, particularly diffuse uptake patterns.

### Echocardiographic Findings

Echocardiography was available for 15 patients. Four patients with negative cardiac FDG PET/CT had normal echocardiographic findings, including left ventricular ejection fraction (LVEF), left ventricular motion, and cardiac chamber and valve assessments. Similarly, five patients with focal cardiac FDG uptake had normal echocardiography.

Among the six patients with diffuse cardiac FDG uptake who underwent echocardiography, only two had normal echocardiographic findings, whereas the remaining four had decreased LVEF, ranging from mild to moderate systolic dysfunction, accompanied in some cases by regional wall motion abnormalities, diastolic dysfunction, pulmonary hypertension, or abnormal septal motion related to conduction disease.

Other key echocardiographic parameters included global longitudinal strain (GLS) and E/A ratio. Patients with diffuse FDG uptake exhibited worse GLS values compared with patients without cardiac involvement (−14.8 ± 0.7% vs. −20.4 ± 1.1%, *p* = 0.010) and compared with patients with focal uptake (−14.8 ± 0.7% vs. −19.2 ± 1.1%, *p* = 0.004). In addition, E/A ratios were lower in patients with cardiac sarcoidosis than in patients without cardiac involvement (0.9 ± 0.2 vs. 1.1 ± 0.1), although this difference was not statistically significant in exploratory analysis. Exploratory correlation analysis demonstrated that higher cardiac SUVmax values were associated with worse GLS values on echocardiography (Spearman's rho = 0.56, *p* = 0.029).

### Cardiac MRI Findings

Nine patients underwent cardiac MRI. Among three with negative cardiac FDG PET/CT, two had normal MRI results, whereas the third showed increased signal with delayed enhancement in the epicardial layer of the mid and apical inferior and lateral left ventricular walls, suggestive of old myocarditis, with LVEF 50% and right ventricular ejection fraction 49%.

Among patients with focal cardiac FDG uptake, cardiac MRI commonly demonstrated patchy or subendocardial late gadolinium enhancement (LGE) corresponding to areas of FDG uptake, sometimes accompanied by ventricular hypokinesia, wall thickening, aneurysmal change, or mildly reduced ventricular function. Among patients with diffuse FDG uptake, MRI findings ranged from absent LGE despite avid FDG uptake to extensive epicardial, transmural, or mid-myocardial LGE involving the interventricular septum and inferolateral left ventricular wall, often associated with reduced LVEF and regional wall motion abnormalities.

**Fig. 1:**
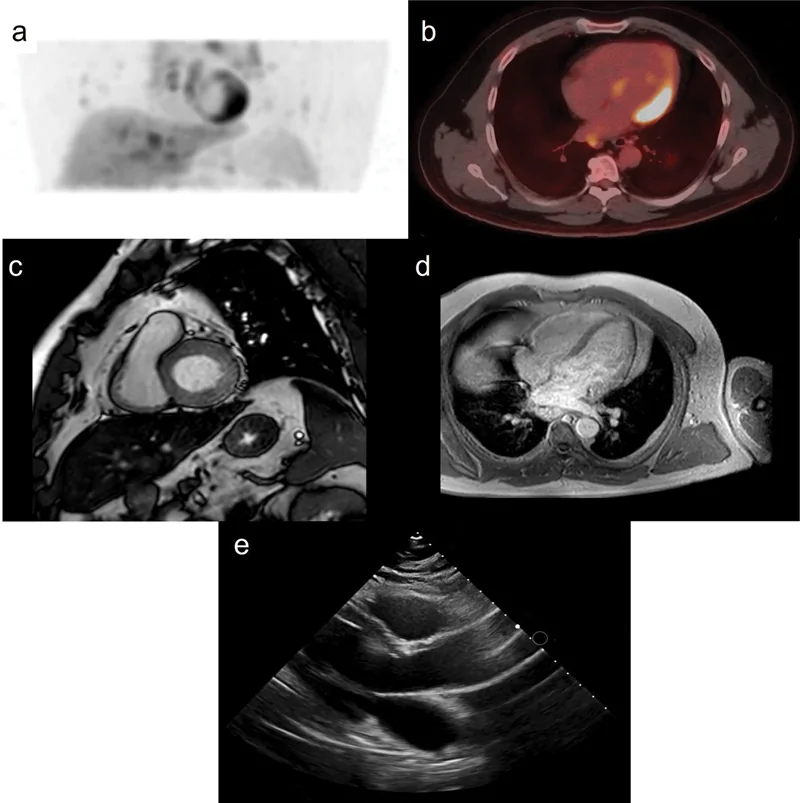
Multimodal imaging of cardiac sarcoidosis in a 41-year-old male patient. ( **a, b** ) Fluorine-18 fluorodeoxyglucose cardiac positron emission tomography/computed tomography images showing increased uptake in the left ventricular myocardium, most marked in the inferior lateral wall at the base. ( **c, d** ) Cardiac magnetic resonance images showing late gadolinium enhancement in the epicardial and mid-myocardial layers of the basal and inferolateral segments of the left ventricle. ( **e** ) Transthoracic echocardiogram demonstrating normal cardiac anatomy and function.

Among the nine patients who underwent both echocardiography and cardiac MRI, LVEF values were consistently lower on cardiac MRI than on echocardiography, with a mean absolute difference of 6.7% based on echocardiographic range midpoints, despite both assessments being performed on the same day or within 1 to 2 weeks of each other. Fig. 1 illustrates FDG PET/CT, MRI, and echocardiographic findings in a patient with cardiac sarcoidosis.

### Treatment and Follow-Up Outcomes

Eighteen patients received prednisone for cardiac or systemic sarcoidosis, often combined with immunosuppressive or biologic agents such as methotrexate, infliximab, adalimumab, hydroxychloroquine, and azathioprine, as determined by the cardiology and rheumatology teams. One patient was treated with deflazacort instead of prednisone, in combination with methotrexate, a therapeutic regimen which was initiated at an outside hospital before presentation to our institution.

Most patients improved clinically following treatment with corticosteroids in combination with immunosuppressive or biologic agents. Device implantation, including permanent pacemaker or implantable cardioverter-defibrillator (ICD) placement, was performed in selected patients with significant arrhythmias or conduction abnormalities. Seventeen patients reported symptom resolution on follow-up. Two patients initially experienced clinical and imaging progression despite therapy, requiring escalation of immunosuppressive treatment. One patient with worsening heart failure and persistent diffuse FDG uptake improved after transition to prednisone and azathioprine, whereas another patient with progressive diffuse FDG uptake despite prednisone and hydroxychloroquine improved following initiation of infliximab.

**Fig. 2:**
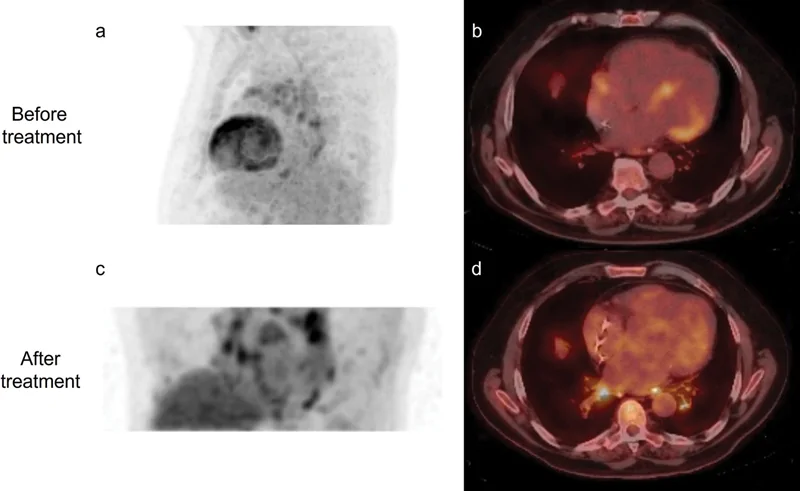
Serial fluorine-18 fluorodeoxyglucose (FDG) positron emission tomography/computed tomography (PET/CT) imaging demonstrating treatment response in cardiac sarcoidosis. ( **a, b** ) FDG PET/CT images of an untreated 71-year-old male patient with cardiac sarcoidosis, showing increased uptake in the left ventricular myocardium mainly at the level of the anterior and inferior walls, sparing the apex. ( **c, d** ) FDG PET/CT images of the same patient following treatment with prednisone and azathioprine as well as implantable cardioverter-defibrillator placement, showing a marked decrease in the extent and activity of the uptake in the left ventricular myocardium at the level of the anterior and inferior walls, consistent with a good response to treatment. There is an increase in the radiotracer uptake within the mediastinal and hilar lymph nodes.

After treatment, follow-up FDG PET/CT demonstrated a significant reduction in cardiac FDG activity, with mean SUVmax decreasing from 9.4 ± 4.2 to 3.8 ± 0.4 ( *p* < 0.001), alongside normalization of myocardial perfusion, confirming a favorable treatment response. Fig. 2 illustrates a representative case before and after treatment, highlighting the decline in FDG uptake and corresponding clinical improvement.

## Discussion

This study reinforces the diagnostic and clinical utility of FDG PET/CT in managing cardiac sarcoidosis, a clinically significant yet often elusive condition. In accordance with previous research, our findings demonstrated characteristic patterns of cardiac FDG uptake, with patients evenly split between diffuse and focal uptake depending on the extent of disease involvement. 5 6 7 8 9 10 Similarly, myocardial perfusion imaging revealed no perfusion defects in patients with diffuse uptake, whereas focal uptake correlated with reduced perfusion in affected areas, a characteristic pattern of cardiac sarcoidosis. 5 8 9 10 Perfusion imaging can help differentiate cardiac sarcoidosis from other conditions; for instance, compression of the microvasculature by active inflammation in sarcoidosis results in a mismatch between perfusion (defect) and FDG imaging (uptake), whereas scarring and fibrosis present as resting perfusion defects without FDG uptake. 5 Furthermore, abnormalities in one or both of the perfusion and FDG images have significant diagnostic, prognostic, and therapeutic implications, such that the combination of abnormal perfusion and abnormal FDG uptake appears to have the worst outcomes. 5

In addition to qualitative analysis, semi-quantitative FDG PET/CT analysis using SUVmax is essential. Although SUVmax is widely used to assess disease burden and activity, no definitive cutoff distinguishes normal myocardium from cardiac sarcoidosis. 5 6 8 10 However, one study identified a cutoff of 4.0, yielding a sensitivity of 97.3% and a specificity of 83.6%. 11 Instead of relying on absolute SUVmax values, current practice evaluates the progression over time to assess disease evolution and treatment response. 5 In our cohort, the mean SUVmax decreased from 9.4 to 3.8 after treatment, a 60% reduction indicating a strong therapeutic response. This highlights the value of serial FDG PET/CT imaging in monitoring disease progression and treatment efficacy.

In regard to treatment, multidisciplinary decisions were made collaboratively by the primary teams following each patient, particularly the cardiology and rheumatology teams, following the American Heart Association (AHA) guidelines for cardiac sarcoidosis management. 12 Notably, both patients with clinical and imaging deterioration despite initial treatment had their therapy escalated according to the tiered approach proposed by these guidelines. 12 One patient, unresponsive to deflazacort and methotrexate, was switched to the first-line steroid prednisone as well as an alternative immunosuppressive agent that had never been used before, azathioprine. The other patient failed treatment with prednisone and hydroxychloroquine, leading to an escalation in treatment with the biologic agent infliximab. Both showed clinical improvement with symptom resolution and reduced cardiac FDG uptake on FDG PET/CT. In addition, patients with significant arrhythmias underwent pacemaker or ICD placement per the 2014 Heart Rhythm Society (HRS) expert consensus statement 13 and 2017 AHA/ACC/HRS guidelines. 14

Our results align with existing literature on echocardiography and its link to cardiac FDG PET/CT in sarcoidosis. Echocardiography may reveal nonspecific findings, such as dilated cardiomyopathy, mild wall thickening, systolic and/or diastolic dysfunction, global hypokinesia, and regional wall motion abnormalities in a noncoronary pattern of distribution. 15 16 However, studies indicate that echocardiography may underdiagnose cardiac sarcoidosis, failing to detect cases with clinical manifestations and positive FDG PET/CT findings. 17 Notably, most patients in our cohort had grossly normal echocardiographic findings, regardless of FDG PET/CT results. In addition, transthoracic echocardiography as the sole screening test has a sensitivity of 25 to 32%, making it a weak screening tool. 18 Consequently, cardiac MRI and FDG PET/CT are recommended despite a negative echocardiogram when symptoms and clinical suspicion persist. 18 19

A particularly useful echocardiographic parameter is GLS, a myocardial deformation index, which is reduced in cases of cardiac sarcoidosis. 20 Research has shown that GLS can be used for diagnosis and prognostication of cardiac sarcoidosis, with a cutoff of −17% showing 94% sensitivity and specificity, and worsening GLS correlating with more severe cardiac outcomes. 20 Our cohort showed a reduced mean GLS of −14.8% in patients with diffuse cardiac sarcoidosis, aligning with this cutoff, compared with normal GLS values of −20.4% in patients without cardiac sarcoidosis and −19.2% in patients with focal disease. Moreover, higher cardiac SUVmax values correlated with worse GLS values, suggesting an association between greater inflammatory burden and impaired myocardial deformation. To our knowledge, no prior studies have reported GLS differences between diffuse and focal cardiac sarcoidosis, warranting further investigation in larger studies. Furthermore, the E/A ratio was numerically lower in patients with cardiac sarcoidosis than in patients without cardiac involvement, aligning directionally with a previous study that showed respective ratios of 0.9 and 1.1 ( *p* = 0.01). 20

The link between FDG PET/CT findings and EKG abnormalities in our study supports growing evidence that FDG PET/CT can help predict arrhythmic complications in cardiac sarcoidosis. This condition is associated with ventricular and supraventricular arrhythmias and conduction abnormalities, such as ventricular tachycardia, ventricular fibrillation, atrial fibrillation, atrial flutter, and atrioventricular block. 10 21 High-degree atrioventricular block is the most common conduction abnormality, likely owing to granulomatous infiltration of the conduction system. 22 Importantly, cardiac sarcoidosis is a major cause of idiopathic Mobitz II and third-degree block, with one study reporting that one-third of young and middle-aged adults with high-degree conduction block were diagnosed with the condition. 22 Accordingly, the 2014 HRS expert consensus statement recommends screening for cardiac sarcoidosis in patients less than 60 years old with unexplained Mobitz II or third-degree atrioventricular block. 13 In concordance with these findings, our cohort included a 55-year-old patient with unexplained complete atrioventricular block who was later diagnosed with cardiac sarcoidosis involving the interventricular septum, possibly affecting the conduction system of the heart as a result, and extending to the free wall of the right ventricle.

Additional evidence linking active cardiac inflammation to conduction disturbances on EKG emerged from other cases in our cohort. Ventricular tachycardia, a common arrhythmia in cardiac sarcoidosis with a significant mortality burden, 23 was documented in one patient via Holter monitoring, with FDG PET/CT showing heterogeneous, patchy FDG uptake in the left ventricle, right ventricle, and interventricular septum. This aligns with research showing similar patterns in electroanatomic mapping of ventricular tachycardia in cardiac sarcoidosis. 23 In addition, five patients had frequent PVCs and/or PACs, one with concurrent left anterior fascicular block and right bundle branch block—conduction abnormalities commonly reported in cardiac sarcoidosis. 24 25 26 27 Notably, these patients had symptomatic PVCs and PACs exceeding 1,000 per day, leading to palpitations and lightheadedness. Although typically benign, such ectopic impulses at high frequencies increase the risk of ventricular dysfunction, atrial fibrillation, cardiomyopathy, and stroke, 28 29 necessitating prompt management. All patients were treated for cardiac sarcoidosis, with resolution of conduction abnormalities and symptoms.

The complementary roles of FDG PET/CT and cardiac MRI in cardiac sarcoidosis are increasingly recognized, with each providing distinct yet interconnected insights. The hallmark of cardiac sarcoidosis on MRI is LGE, 5 observed in most of our patients. This helps distinguish cardiac sarcoidosis from prior infarction, although, as noted in one of our cases, cardiac sarcoidosis can rarely mimic an infarct with subendocardial LGE. 5 However, LGE is not always present; one patient in our cohort had avid FDG uptake on FDG PET/CT but no LGE on MRI, highlighting that cardiac sarcoidosis can exist without LGE. 30 Studies show that LGE-positive patients face higher risks of adverse cardiovascular events, such as arrhythmias and sudden cardiac death. 30 In our cohort, the only patient without LGE had normal EKG findings, whereas all patients with LGE exhibited arrhythmias, including ventricular tachycardia, PVCs, and PACs. Interestingly, LVEF was consistently lower on cardiac MRI than echocardiography, contrary to prior studies showing that echocardiography typically underestimates LVEF compared with cardiac MRI. 31 32 The reason for this discrepancy is unclear, as all cardiac MRIs and echocardiograms followed standardized protocols, were performed either on the same day or within 1 to 2 weeks of each other, and were interpreted by experienced physicians.

Current trends favor combining cardiac MRI and FDG PET/CT to achieve synergistic effects, with MRI assessing structural damage such as myocardial necrosis and fibrosis or scarring, and FDG PET/CT evaluating disease burden and active inflammation. 10 33 34 35 FDG PET/CT offers better sensitivity, whereas cardiac MRI provides higher specificity; therefore, combining the two modalities improves both sensitivity and specificity compared with using either one alone. 10 One study reported sensitivity rates of 85% and 82% for FDG PET/CT and MRI, respectively, whereas hybrid PET/MRI increased sensitivity to 94%. 34 Similarly, another study found high specificity (96%) for co-localized PET/MRI findings. 35 PET/MRI offers deeper disease characterization by assessing both activity and underlying injury in a single scan. 10 33 34 35 Studies demonstrate its superiority in diagnosis, assessment, monitoring, and prognostication of cardiac sarcoidosis, and future advancements may further enhance its clinical implementation through technical innovations. 10 33

The availability of FDG PET/CT positions our institution as a leading center for diagnosing and managing cardiac sarcoidosis in Lebanon and the broader Middle East and North Africa (MENA) region, where access to specialized imaging remains limited. Despite the significant regional burden of sarcoidosis, 36 diagnostic tools for cardiac involvement are not widely accessible, likely resulting in underreporting and delayed intervention. This issue is compounded by the lack of MENA-based studies, creating a critical gap in the literature. Our institution serves as a key resource for early and accurate diagnosis through FDG PET/CT, in line with global trends where FDG PET/CT is now a routine modality incorporated into consensus algorithms for diagnosis and monitoring. 5 13 Beyond contributing to the broader literature, our study highlights the feasibility and efficacy of cardiac FDG PET/CT in resource-limited settings, setting a precedent for expanding this technology in similar health care contexts. Future efforts should focus on regional collaboration to improve FDG PET/CT access, provide standardized training for radiologists and clinicians, and establish diagnostic and therapeutic protocols for the MENA region, ultimately addressing disparities in disease management and patient outcomes.

This study was limited by its retrospective design and small sample size, which may affect the generalizability of the results. The exploratory statistical analyses should be interpreted cautiously because of the limited cohort size and retrospective design, which reduced statistical power and precluded robust multivariable modeling. Moreover, variations in treatment strategies and follow-up durations may have introduced inconsistencies. Larger, prospective studies are needed to validate the diagnostic accuracy and clinical impact of FDG PET/CT in cardiac sarcoidosis, particularly in comparison with other imaging modalities. Future research should also focus on standardizing imaging protocols, establishing quantitative FDG PET/CT biomarkers for disease activity and treatment response, and investigating its prognostic value in predicting long-term cardiac outcomes. Furthermore, as FDG PET/CT becomes more accessible, particularly in resource-limited settings, broader regional analyses may reveal interesting trends in the epidemiology, clinical presentation, and imaging findings of cardiac sarcoidosis in the MENA population, informing tailored treatment guidelines and advancing personalized medicine. Expanding access to FDG PET/CT, especially for cardiac applications, will be essential for enabling more accurate and timely diagnosis, improved treatment planning, and better patient monitoring across a wider demographic.

## Conclusion

This study reinforces the integral role of FDG PET/CT in diagnosing and managing cardiac sarcoidosis, particularly when integrated with MRI, echocardiography, and EKG. FDG PET/CT not only detects active inflammation but also guides treatment decisions, predicts arrhythmic risk, and monitors therapeutic response. Notably, this study provides new insights by demonstrating the feasibility and effectiveness of FDG PET/CT in a resource-limited setting, an aspect largely underexplored in the literature. It highlights the potential of FDG PET/CT to improve early diagnosis and personalized management in the MENA region, where access to advanced imaging is often limited. Expanding FDG PET/CT access could transform clinical decision-making, enabling earlier intervention, optimized treatment strategies, and better patient outcomes.
